# Anti-inflammatory Therapies for Ischemic Heart Disease

**DOI:** 10.1007/s11886-025-02211-0

**Published:** 2025-02-19

**Authors:** Tillmann Muhs, Senka Ljubojevic-Holzer, Susanne Sattler

**Affiliations:** 1 Department of Pharmacology, Otto-Loewi Research Center, Medical University of Graz, Neue Stiftingtalstraße 6, 8010 Graz, Austria; 2 Department of Cardiology, LKH Univ. Klinikum Graz, Medical University of Graz, Auenbruggerplatz 15, 8036 Graz, Austria; 3 National Heart and Lung Institute, Imperial College London, Hammersmith Campus, Du Cane Road, London, W12 0NN UK

**Keywords:** Inflammation, Ischemic heart disease, Colchicine, SGLT2i, IL-6, NLRP3 inflammasome, Immunomodulation, NLRP3 inhibition, Interleukin therapy

## Abstract

**Purpose of Review:**

The inclusion of immunomodulatory strategies as supportive therapies in ischemic heart disease (IHD) has garnered significant support over recent years. Several such approaches appear to be unified through their ultimate target, the NLRP3 inflammasome. This review presents a brief update on immunomodulatory strategies in the continuum of conditions constituting ischemic heart disease and emphasising on the seemingly unifying mechanism of NLRP3 activation as well as modulation across these conditions.

**Recent Findings:**

The NLRP3 inflammasome is a multiprotein complex assembled upon inflammatory stimulation, causing the release of pro-inflammatory cytokines and initiating pyroptosis. The NLRP3 pathway is relevant in inflammatory signalling of cardiac immune cells as well as non-immune cells in the myocardium, including cardiomyocytes, fibroblasts and endothelial cells. In addition to a focus on clinical outcome and efficacy trials of targeting NLRP3-related pathways, the potential connection between immunomodulation in cardiology and the NLRP3 pathway is currently being explored in preclinical trials. Colchicine, cytokine-based approaches and SGLT2 inhibitors have emerged as promising agents. However, the conditions comprising IHD including atherosclerosis, coronary artery disease (CAD), myocardial infarction (MI) and ischemic cardiomyopathy/heart failure (iCMP/HF) are not equally amenable to immunomodulation with the respective drugs. Atherosclerosis, coronary artery disease and ischemic cardiomyopathy are affected by chronic inflammation, but the immunomodulatory approach to acute inflammation in the post-MI setting remains a pharmacological challenge, as detrimental and regenerative effects of myocardial inflammation are initiated in unison.

**Summary:**

The NLRP3 inflammasome lies at the center of cell mediated inflammation in IHD. Recent trial evidence has highlighted anti-inflammatory effects of colchicine, interleukin-based therapy as well as SGLT2i in IHD and that the respective drugs modulate the NLRP3 inflammasome.

## Introduction

IHD consists of the continuum of atherosclerosis, coronary artery disease (CAD), myocardial infarction (MI) and ischemic cardiomyopathy (iCMP), which are characterised by a mismatch of oxygen demand and supply. Global mortality statistics ranked by disease are led by IHD and the demographic shift to an aging society is anticipated to exacerbate this further highlighting the need for better therapeutic strategies [1].

**Fig. 1:**
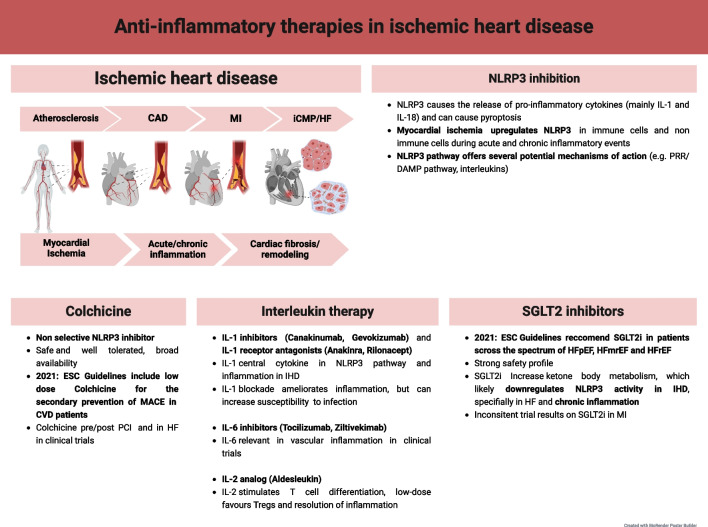
Anti-inflammatory therapies in ischemic heart disease. The continuum of conditions constituting IHD is driven by acute and chronic inflammatory events following myocardial ischemia. NLRP3 inhibition contributes to anti-inflammatory effects observed in colchicine, interleukin-based therapy and SGLT2i. Key: Ischemic heart disease (IHD), coronary artery disease (CAD), myocardial infarction (MI), ischemic cardiomyopathy, (iCMP), heart failure (HF), NOD-, LRR- and pyrin domain-containing protein 3 (NLRP3), interleukin (IL), pattern recognition receptors (PRR), damage associated molecular pattern (DAMP), sodium-glucose-like-transporter 2 inhibitor (SGLT2i), major adverse cardiac events (MACE), cardiovascular disease (CVD), percutaneous coronary intervention (PCI), European Society of Cardiology (ESC), heart failure preserved ejection fraction (HFpEF), heart failure mildly reduced ejection fraction (HFmrEF), heart failure reduced ejection fraction (HFrEF)

Today, atherosclerosis is understood as a chronic inflammatory disease preceding CAD, the acute event of MI, as well as iCMP, the late result of myocardial ischemia [2]. In MI due to acute occlusion of coronary arteries, extensive areas of the myocardium become ischemic, and die, initiating an acute inflammatory response [3]. The area at risk for necrosis may be salvaged by timely reperfusion through percutaneous coronary intervention (PCI), but the restoration of blood-flow to the previously ischemic myocardium results in ischemia/reperfusion injury (I/RI), which is itself a powerful inflammatory stimulus [4, 5]. Excessive levels of myocardial inflammation promote cardiac fibrosis, remodelling and the manifestation of iCMP, which clinically presents as HF (Fig. 1) [3, 5]. While the mechanistic link between ischemia and inflammation is established, the recent CANTOS trial (2017) also demonstrated clinically, that ameliorating inflammation in atherosclerotic patients has potential to reduce the incidence of major adverse cardiac events (MACE) [6].

ESC guidelines on CAD, MI and HF recommend several families of drugs, including nitrates, β-blockers, calcium channel blockers and NO donors to manage myocardial ischemia [7–9]. Yet, few of those control both myocardial ischemia and the resulting acute and chronic inflammation as well as the incidence of MACE. This article aims to present an update on recent developments in clinical anti-inflammatory strategies in IHD.

## The NLRP3 Inflammasome at the Centre of Inflammatory Pathways in IHD

The exploration of anti-inflammatory mechanisms in cardiology and IHD has led to the NLRP3 inflammasome at the centre of vascular and myocardial inflammation [10]. Inflammasomes are intracellular multi-protein complexes, assembled upon inflammatory stimulation [10, 11]. It was thought initially that leukocytes are the primary cell type to express NLRP3, but it can also be upregulated in non-immune cells during inflammatory processes [12, 13]. Importantly, this includes cardiomyocytes and cardiac fibroblasts, which have been shown to express NLRP3 components, although NLRP3 signalling in non-immune cells of the myocardium is less well understood [14–16].

A simplified model of NLRP3 activation consists of (a) initial priming through nuclear factor kappa B (NF-κB) mediated signalling, which increases transcription of NLRP3 components and (b) activation upon component assembly [11, 17]. A broad range of intra- and extracellular inflammatory stimuli increases NLRP3 activity, including lysosomal rupture, mitochondrial dysfunction and excessive ROS production, DAMP/PAMP recognition via the PRR/TLR-receptor pathway and cytokine signalling (mainly IL-1, IL-6 and IL-18) [10, 17]. Downstream inflammatory effects are primarily realised through caspase activation, cleavage of IL-1β and gasdermin D inducing membrane pore formation and enforcing pyroptosis [17–19].

Mitochondrial dysfunction, oxidative stress and dysregulation of ion homeostasis of cardiomyocytes during myocardial ischemia are well documented [20–23]. Cardiomyocytes may tolerate brief episodes of ischemia, but prolonged ischemia causes pyroptosis and the release of intracellular contents to the extracellular milieu [24, 25]. Cellular debris is recognised by the PRR-pathways of cardiac immune and non-immune cells, which start the inflammatory cascade orchestrated by NLRP3 activation, including leukocyte recruitment and activation of the adaptive immune response [11, 26].

Some immune cells can escape NLRP3 induced pyroptosis through self-mediated NLRP3 inhibition. Dendritic cells can supress NLRP3 activation through the expression of transcription factors IRF4/8, ultimately preventing pyroptotic cell death and preserving the priming of T cells [27, 28]. This mechanism of inherent NLRP3 suppression is proposed to be relevant in other immune cells too, including macrophages and Tregs [29, 30]. Foxp3, a central transcription factor in Treg differentiation, controls the expression of IRF4/8, which is proposed to contribute to the immunosuppressive function of Tregs [31–33]. As Tregs are relevant in regulating vascular and myocardial inflammation, this indicates that targeting Foxp3 and IRF4/8 could modulate NLRP3 activity and the Treg response [31, 34, 35]. The inherent ability of the respective immune cells to self-regulate NLRP3 expression could serve as a role model for future immunomodulatory strategies for NLRP3 inhibition.

The NLRP3 pathway thus offers several potential targets for immunomodulation in IHD [17, 36–40]. With the NLRP3 inflammasome being relevant in immune and non-immune cells of the vasculature and myocardium, modulation of low-grade chronic inflammation underlying atherosclerosis, coronary artery disease and remodelling in ischemic cardiomyopathy could be achieved with targeted NLRP3 inhibition [12, 13, 16]. With initial steps towards precision medicine in IHD [41–43], guided by the development of nano-particles for cell-specific drug delivery [44], a combined approach of NLRP3 inhibition in selected cell populations could reduce doses required to achieve therapeutic immunomodulation and thereby the susceptibility to infection [45]. Therefore, the development of new NLRP3 inhibitors [46–50], as well as elucidation of the effects of established drugs on the NLRP3 inflammasome, are of great interest going forwards [51].

## Colchicine

Colchicine is an anti-inflammatory drug previously used in patients with gout and pericarditis, which inhibits leukocytes, primarily neutrophils, through the promotion of microtubule depolymerisation [52]. Further, neutrophils release neutrophil extracellular traps (NET) in a process called NETosis, which is relevant in inflammatory events in atherosclerotic lesions as well as MI, however the effects of colchicine on NETosis remain unclear [53–56]. Although these mechanisms are relevant in IHD, colchicine has additional anti-inflammatory effects beyond inhibition of leukocyte function, which are the subject of interest in current immunomodulatory trials [57].

Colchicine modulates several steps of the inflammatory response, including the inhibition of cytokine synthesis (interleukins, TNF, leukotrienes, prostaglandins and TXA2), and leukocyte attraction and migration (P-selectin expression) [52]. Many of these anti-inflammatory effects are caused by the inhibition of the NLRP3 inflammasome in active immune cells during vascular and myocardial inflammation [36]. The results from the COLCOT trial (2019) demonstrated that daily colchicine significantly reduces the risk of ischemic cardiovascular events in patients post-MI [58]. Shortly after, the LoDoCo2 trial (2020) used the same dosing regime to achieve a significant reduction of cardiovascular events in patients with chronic coronary artery disease [59]. Since then, ESC guidelines recommend low-dose colchicine therapy for the secondary prevention of cardiovascular events in patients with CVD [60]. Further confirmation that low-dose colchicine has beneficial effects on inflammation and MACE in IHD was provided by two separate systematic reviews by Ebrahimi et al. (2023) and Fiolet et al. (2024) [61, 62].

Substantial efforts have been made to evaluate, whether colchicine administration would also yield beneficial effects on patient outcomes pre- or post PCI, but the current state of pre- or post-procedural colchicine remains unclear. Study results lack comparability as sample sizes were limited, dose and time regimes varied, and different outcome measurements were used between individual trials [63–66]. The POPCORN, COLCHICINE-PROTECT and COL BE PCI trials are underway and will provide more insight into colchicine effects in the setting of PCI (Unique identifier: NCT05618353, NCT05739929, NCT06095765) [67]. It remains to be noted, that inhibiting inflammation early after MI, and subsequent PCI, should be approached carefully, considering the potentially detrimental effects of disrupting wound healing and scar formation following MI [68–70].

The potential use of colchicine to modulate the interplay between inflammatory pathways and cardiac fibrosis and remodelling in HF is also being investigated. The COLICA trial (2024) demonstrated that colchicine significantly reduces CRP and IL-6 serum concentrations following acute HF, but effects on NT-proBNP levels and new HF diagnoses were not significant [71]. A conclusion on benefits of colchicine in HF as well as appropriate treatment regimes is yet to be made [72]. The COLT-HF trial will provide further insights in the potential benefits of colchicine for patients with HF due to IHD (Unique identifier: NCT05873881).

Finally, colchicine is widely available and safe. Low-dose colchicine treatment is generally well-tolerated, besides mild diarrhoea upon treatment initiation [73]. However, IHD is associated with an often multimorbid patient population, in which drug administration can be complicated by chronic kidney disease and pharmacological drug interactions. Concurrent treatment with selected antibiotics, antimycotics and antiviral drugs should be approached carefully.

## Interleukins

Interleukins (IL) are a heterogenous group of cytokines produced by immune and non-immune cells in response to inflammatory stimuli. They mediate immune responses by regulating adhesion, migration, proliferation and maturation of immune cells [74]. While most initial trials focused on blocking pro-inflammatory interleukins, recent explorations have moved towards boosting anti-inflammatory properties of cytokines in IHD.

IL-1 is one of the most potent intrinsic stimulators of inflammation, and in IHD its production and release is inseparable of NLRP3 activity in active immune and non-immune cells [37, 75]. Up-regulation of IL-1 in the ischemic myocardium is followed by the onset of acute inflammation, which triggers cardiac remodelling and its long-term consequence iCMP [76–78]. The CANTOS (2017) trial demonstrated that the IL-1β inhibitor Canakinumab can significantly reduce the rate of recurrent cardiovascular events in atherosclerotic patients independent of lipid-lowering therapy, underscoring the relevance of inflammation and cytokine inhibition in atherosclerosis and subsequent IHD [6, 79]. In preclinical models of MI and HF the IL-1 inhibitor Gevokizumab has achieved similar cardiovascular effects emphasising again the potential of IL-1 modulation in IHD [80, 81].

Modulating the IL-1 pathway has seen further approaches using the IL-1 receptor antagonist Anakinra. A series of trials (VCU-ART 1–3) evaluated the effects of Anakinra post-MI and demonstrated beneficial effects on the inflammatory response and heart function [82–84]. A pooled meta-analysis on the VCU-ART trials outlined that Anakinra significantly reduced the incidence of new-onset HF or hospitalisation for HF and a 4th iteration of the trial series is underway (VU-ART4; Unique identifier: NCT0517782) [85]. 

Myocarditis is commonly caused by viral infection and should be viewed separately from sterile inflammation in IHD, but the underlying inflammatory pathways may be shared, as the death of virus infected cardiomyocytes also releases DAMPs, which initiates myocardial inflammation and subsequent cardiac remodelling via the PRR-/NLRP3 pathway [86–88]. Consequently, the results of the ARAMIS trial (2023) on patients with acute myocarditis could add to insights of pharmacological IL-1 modulation in the inflamed myocardium using Anakinra [89, 90].

Altogether, the relevance of the IL-1 pathway in IHD has been demonstrated by a series of trials, which have catalysed research on inflammatory processes in IHD. While providing an important proof-of-concept that inflammation in atherosclerosis is a clinically relevant and targetable factor, the FDA rejected approval of Canakinumab to reduce cardiovascular inflammation and IL-1i/Ra are not indicated to treat CVD/IHD. Although IL-1 inhibition reduces leukocyte and neutrophil count, cases of severe neutropenia are rare [6]. Interestingly, the main safety concern was not an increased risk of infection itself, but a delay to initiation of treatment due to IL-1 inhibition masking clinically important signs of infection (i.e. fever, swelling or redness), thus resulting in fatal infection [6, 91]. Despite, IL-1 modulation remains an excellent tool to explore the intricacies of the inflammatory response and early steps towards tissue-specific drug delivery are taken using the IL-1 inhibitor Gevokizumab and the immunosuppressant methotrexate [92, 93]. These strategies are based on loading immunomodulatory agents into LDL-like nanoparticles or utilising microparticles as carriers (i.e. platelet-membrane), optimising local drug delivery and reducing adverse effects of systemic immunomodulation [94–96]. This marks an important development towards precision medicine as classic systemic immunomodulation always carries the risk of increased susceptibility to infectious disease.

IL-6 has pro-inflammatory effects in cardiovascular disease. Its release is orchestrated by NLRP3 activity and is reinforced, but not dependent on IL-1 signalling [97–99]. IL-6 contributes to the development of IHD through modulating the inflammatory response of immune and non-immune cells [100, 101]. Further, endothelial and vascular smooth muscle cells may express IL-6 receptors dependent on IL-1 activity, indicating the relevance of IL-6 in atherosclerotic and subsequent ischemic heart disease [102–104]. Recently, the IL-6 receptor antagonist Tocilizumab gained relevance as a treatment of cytokine-release-syndrome during COVID-19 infection [105, 106]. While trials have highlighted the anti-inflammatory benefits of Tocilizumab in cardiovascular patients, direct effects on improving clinical outcomes post-MI have not been convincingly demonstrated [107–109]. Further, treatment with Tocilizumab can increase LDL and cholesterol serum concentrations, which alongside inflammation is a known risk factor for CVD [110, 111]. However, similar to IL-1 inhibition, treatment with Tocilizumab can increase the susceptibility to infection, and the EMA approval of Tocilizumab is limited to rheumatoid conditions and cytokine-release-syndrome [111–113]. Research on modulating the IL-6 pathway has continued in the RESCUE trial, which demonstrated that Ziltivekimab, an IL-6 monoclonal antibody, reduces biomarkers of inflammation in atherosclerotic patients [114]. A post-hoc analysis extended on these results, suggesting Ziltivekimab can disrupt multiple atherogenic inflammatory pathways [115]. The ongoing ZEUS trial will further elaborate on the anti-inflammatory effects of Ziltivekimab in cardiovascular and cardiorenal patients (ZEUS; Unique identifier: NCT05021835) [97, 116]. So far, modulation of IL-6 promises to become an effective tool to combat vascular inflammation in atherosclerosis and CAD, which precedes MI and HF/iCMP.

While inhibiting IL-1/6 can reduce inflammation, the increased susceptibility to infections makes this strategy difficult to navigate in patients. Immunomodulation aimed at IL-2 could offer a more refined therapeutic strategy compared to IL-1/6 inhibition. In IHD, CD4 ^+^ T cells are pivotal players of immunomodulation [117, 118]; importantly, pro-inflammatory effector T cells (Teff) expand in IHD [119], while numbers of regulatory T cells (Tregs) decrease following MI [120, 121]. Preclinical data highlight that Tregs have atheroprotective function and contribute to myocardial healing and repair [31, 122–126]. In MI, auto-antigen-dependent and antigen-independent T cell activation causes the release of IL-2 and autocrine stimulation of T cell proliferation [123, 127]. Further, CD4 ^+^ T cell differentiation may be dependent on NLRP3 expression, which has been suggested to be controlled by IL-2 [29]. In the LILACS trial (2022) low-dose IL-2 was safe and expanded Treg, but not Teff populations, confirming that IL-2-controlled differentiation of CD4 ^+^ T cells is dose dependent [128]. The trial used Aldesleukin, a recombinant analog of IL-2. The IVORY trial is ongoing and designed to evaluate the effects of low-dose IL-2 on vascular inflammation in patients with acute coronary syndrome [129]. While the IL-2 approach to ameliorate inflammation in IHD is in its early stages, current trial results and preclinical data highlight the safety advantage of promoting anti-inflammation and regeneration without affecting immune defence against infections.

## SGLT2 Inhibitors

Despite not strictly considered an anti-inflammatory drug, sodium-glucose-like-transporter-2 inhibitors (SGLT2i) deserve a mention in this context. While originally used as oral antidiabetics, they have demonstrated multiple benefits in cardiovascular patients, and some of those may in fact be linked to immunological effects. In 2015, results from the EMPA-REG OUTCOME trial (2015) were followed by a meta-analysis by Zelniker et al. (2019) reviewing the cardiovascular benefits of SGLT2i in diabetic patients with concurrent CVD demonstrated in several trials [130–133]. The compounding line of evidence led to the ESC including SGLT2i as first-line therapy in diabetic patients with pre-established CVD [134]. The benefit of SGLT2i in patients with HF and reduced, mid-range and preserved EF independent of diabetes was established by subsequent trials [7, 135–137].

The primary effect of SGLT2i is thought to occur through renal SGLT2 inhibition and subsequent induction of glucosuria and osmotic diuresis – this benefits serum levels of glucose and decreases cardiac pre- and afterload [138, 139] Consequently, a cardioprotective effect due to improved cardiac hemodynamics from SGLT2i should not be surprising [140]. However, SLGT2i show extra-renal effects beyond glycemic control and optimised cardiac hemodynamics. Cardiac cells, including cardiomyocytes, fibroblasts, endothelial and smooth muscle cells, to a large extend, are devoid of SGLT2, therefore, cardiovascular anti-inflammatory effects of SGLT2i is suggested to be independent of SGLT2 expression [141–144]. However, myocardial cells have been found to transiently express SGLT2 under ischemic conditions [145]. Research on the mechanisms underlying anti-inflammatory effects of SGLT2i is ongoing, but initial preclinical data indicate a modulation of the NLRP3 axis in cardiac cells leading to reduced inflammatory factors including IL-1β, IL-6, IL-18 and TNF [146, 147]. Consequently, downstream immunomodulatory effects are likely to be multifactorial but include enhanced anti-inflammatory macrophage polarisation, attenuation of cardiac fibrosis and reduced ROS production [148–150]. Importantly, the EMMY trial (2023) highlighted the potential mechanism of NLRP3 inhibition using the ketone metabolite beta-hydroxybutyrate [151, 152], which has been demonstrated to be increased early post-MI and is suggested to be relevant in the development of HF [153]. This is in line with findings, suggesting SGLT2i modulate myocardial metabolism to increase utilisation of ketone bodies and fatty acids under ischemic conditions and post-MI [142, 154, 155]. With NLRP3 inhibition recently receiving attention, this further underscores an immunomodulatory function of SGLT2i and potential clinical relevance of ketone bodies in IHD [152, 156–158].

In principle, the suggested immunomodulatory mechanisms of SGLT2i could also prove beneficial in patients with previous MI. Sayour et al. (2021) concluded that SGLT2i can limit and reduce infarction size in animal models and preclinical data has been followed-up by several clinical trials evaluating the effects of SGLT2i in the post-MI setting [159]. The EMMY (2022) trial demonstrated that SGLT2i treatment in patients with previous MI had beneficial effects on cardiac function, NT-proBNP serum concentrations and cardiometabolic outcomes [151]. Results from the DAPA-MI trial (2023) were mixed; SGLT2i post-MI had similar beneficial effects on cardiometabolic outcomes compared to patients from the EMMY trial, but did not impact the composite of cardiovascular death or hospitalisation for HF [158]. In the EMPACT-MI trial (2024), SGLT2i inhibition did not reduce the risk of first hospitalisation for HF or death from any cause in patients post-MI [160]. Notably, the EMMY trial was not designed to assess clinical outcomes, but cardiac function and biomarkers of HF [151]. However, the EMMY and DAPA-MI trial demonstrated similar results on the effects of SGLT2i on cardiometabolic outcomes, emphasising the potential immunomodulatory mechanism of ketone bodies in myocardial inflammation resulting from ischemia and NLRP3 activation [142, 151, 152, 156, 158]. SGLT2i had no significant impact on clinical outcomes in both the DAPA-MI and the EMPACT-MI trial [158, 160]. This raises the question, whether pathophysiological processes post-MI are equally amenable to SGLT2i immunomodulation across phases of MI healing, in which each phase (inflammatory, proliferative and maturation) is dominated by specific immune cell populations and inflammatory pathways [160, 161]. Further, treatment duration between the respective trials varied and cardiometabolic benefits were reported for EMMY and DAPA-MI trial (EMMY 26 days, DAPA-MI 1 year, EMPACT-MI 14 days), suggesting immunomodulatory mechanisms of SGLT2i are more effective during later phases of MI healing. From an immunological perspective, the transition from late MI healing to iCMP/HF can be subtle and is dominated by adaptive immunity and processes of cardiac remodelling [161]. Here, immunomodulation using SGLT2i may be most effective, which would be in line with their proven benefits in patients with HF [162, 163].

## Conclusion and Future Directions of Immunomodulation in IHD

Pharmacological NLRP3 inhibition appears to be a logical conclusion to recent findings on cell mediated inflammation, which put the NLRP3 inflammasome at the centre of processing inflammatory signalling on one hand and orchestrating the release of inflammatory cytokines on the other. As the inflammatory response in IHD is continuously being mapped out, potential targets to achieve NLRP3 inhibition, subsequently controlling vascular and myocardial inflammation, are identified. This includes a potential immunomodulatory strategy to target NLRP3, Foxp3 and IRF4/8 in immune cells to achieve anti-inflammatory effects in IHD, although little data on concrete approaches is available yet. At this time the immunomodulatory use of colchicine, strategies based on modulating IL pathways and SGLT2i are the most promising approaches with evidence from preclinical studies and clinical trials.

Importantly, the release of interleukins is dependent on NLRP3 activity. While up to 40 different interleukins have been identified, IL-1, IL-2 and IL-6 are promising immunomodulatory targets in IHD. Here, IL-1 inhibition received most attention with trials on Canakinumab and Anakinra, though challenges related to infection susceptibility remain a hurdle for clinical adoption in IHD. Emerging approaches in precision medicine, such as localized drug delivery systems, offer hope for overcoming these limitations. Low-dose IL-2 treatment with Aldesleukin may offer an elegant solution as it can promote inflammation controlling qualities of the adaptive immune system without affecting host defence or regenerative functions. Initial results on IL-6 inhibition in IHD using Tocilizumab were not convincing, but IL-6 inhibition could still prove to be valuable in IHD with trials investigating Ziltivekimab.

While targeting interleukins is pathway-specific, colchicine outside its use in gout has recently been introduced as an immunomodulatory drug in IHD due to its properties as a non-selective NLRP3 inhibitor. ESC guidelines have included colchicine in the recommended drug repertoire to reduce cardiovascular events in post-MI and chronic coronary artery disease patients, with ongoing trials poised to further refine its use in the acute and chronic phases of IHD management. Notably, colchicine offers the advantage of safety, tolerability, availability and cost-efficiency, and with the burden of a growing number of cardiovascular patients in mind, this makes colchicine a well-suited choice for day-to-day management of patients with atherosclerosis and CAD.

SGLT2i have evident benefits in cardiology. The recent focus on immunomodulation has led to the exploration of direct anti-inflammatory mechanisms of SGLT2i on the heart. Preclinical evidence as well as selective data on cardiometabolic outcomes from recent clinical trials support the role of SGLT2i in attenuating inflammation and remodelling through mechanisms involving the NLRP3 inflammasome and ketone body metabolism. Combining research on diabetic and cardiovascular patients from the past 15 years, trials have collectively provided a strong safety profile, and SGLT2i are included in ESC guidelines across the spectrum of HF.

In conclusion, NLRP3 inflammasome-centered strategies including colchicine, interleukin-directed therapies and possibly SGLT2 inhibitors hold great promise as immunomodulators targeting cardiovascular inflammation. Ongoing efforts of their refinement through emerging strategies from precision medicine promise to improve safety profiles for effective use in the cardiology clinicals.

## Key References

- Fiolet ATL, Poorthuis MHF, Opstal TSJ, Amarenco P, Boczar KE, Buysschaert I, et al. Colchicine for secondary prevention of ischaemic stroke and atherosclerotic events: a meta-analysis of randomised trials. eClinicalMedicine. 2024;76
  - This meta-analysis underscores the evidence indicating the anti-inflammatory potential of colchicine in IHD and CAD. Low-dose colchicine is effective in reducing MACE in patients with CVD and has been ESC approved to for the secondary prevention of MACE.
- Toldo S, Mezzaroma E, Buckley LF, Potere N, Di Nisio M, Biondi-Zoccai G, et al. Targeting the NLRP3 inflammasome in cardiovascular diseases. Pharmacol Ther. 2022;236:108053.
  - This review highlights the diverse approaches to NLRP3 inhibition, including established methods and others that have demonstrated anti-inflammatory effects in CVD. Furthermore, it outlines the connection between the anti-inflammatory mechanisms of NLRP3 inhibition and their clinical effects in CVD.
- Zhao TX, Sriranjan RS, Tuong ZK, Lu Y, Sage AP, Nus M, et al. Regulatory T-Cell Response to Low-Dose Interleukin-2 in Ischemic Heart Disease. NEJM Evidence. 2022;1(1):EVIDoa2100009.
  - This study demonstrates an elegant approach to interleukin-based strategies in IHD as low-dose IL-2 causes dose dependent Treg differentiation and promotes resolution of inflammation without affecting the immune systems host-defence. This represents an improvement over classical IL-1 inhibition, which has been critically reviewed due to its association with an increased susceptibility to infection.

## Data Availability

No datasets were generated or analysed during the current study.
